# The prognostic significance of Albumin-to-Alkaline Phosphatase Ratio in upper tract urothelial carcinoma

**DOI:** 10.1038/s41598-018-29833-5

**Published:** 2018-08-17

**Authors:** Ping Tan, Nan Xie, Jianzhong Ai, Hang Xu, Huan Xu, Liangren Liu, Lu Yang, Qiang Wei

**Affiliations:** 1 Department of Urology, Institute of Urology, West China Hospital, Sichuan University, Chengdu, China; 2 Department of Emergency, West China Hospital, Sichuan University, Chengdu, China; 3 Department of Pathology, West China Hospital, Sichuan University, Chengdu, China

## Abstract

To assess the prognostic impact of pretreatment albumin-to-alkaline phosphatase ratio (AAPR) in patients with upper tract urothelial carcinoma (UTUC), the data of 692 patients, operated between 2003 and 2016 in our center, were retrospectively assessed. The threshold of AAPR was defined as 0.58 by using the receiver-operating curve analysis. Overall survival (OS), cancer-specific survival (CSS), and recurrence-free survival (RFS) were evaluated using the Kaplan-Meier method. And the univariate and multivariate Cox’s regression models were performed to identify independent prognostic predictors. The results showed that AAPR <0.58 was significantly related to higher pT stage and grade, concomitant variant histology, anemia and larger tumor size. Additionally, patients with a lower AAPR had an inferior survival outcomes than those with an AAPR ≥0.58 (all P < 0.001). Multivariate analysis suggested that the lower AAPR was also an independent risk factor for poor OS (HR 1.587, 95%CI: 1.185–2.126; P = 0.002), CSS (HR 1.746, 95%CI: 1.249–2.440; P = 0.001), and RFS (HR 1.337, 95%CI: 1.027–1.739; P = 0.031). Moreover, subgroup analysis demonstrated the lower AAPR was related to worse prognosis in high-grade UTUC patients; but in those with low-grade disease, no relationship between them was observed. In conclusion, our results found that the decreased AAPR was independently related to poor survival outcomes in UTUC patients. Using the AAPR for subclassification of high-grade UTUC seems to further identify a poor prognostic group and contribute to clinical decisions making.

## Introduction

Upper tract urothelial carcinomas (UTUCs) accounts approximately 5% of urothelial carcinomas (UCs), including renal pelvicalyceal and ureteric urothelial carcinomas^1^. Due to the exposure of aristolochic acid in Chinese herbs and arsenic in water, the incidence of UTUC in Asian countries, especially in Taiwan district, was found to be much higher than that in western countries^1^. The data shows approximately 60% of UTUC are invasive at the time of diagnosis, and their prognosis are relatively poor^1^. At present, radical nephroureterectomy is still the standard care for treatment of patients with invasive, non-metastatic UTUC^1^. However, there remains a high risk of local or distant recurrence with nodal disease found in up to 30% of patients after RNU^2^. Thus recently, increasing number of people raised interests in the pre- and post-operative prognostic predictors. Tumor stage and grade are the best-established prognostic predictors in UTUC, however, most of the clinicopathological features can be accurately determined after surgical excision. Identifying preoperative prognostic markers could help stratify patients with worse outcomes or with a high risk of recurrence who may possibly benefit from neoadjuvant chemotherapy. At the moment, some blood-based markers have been established, including hemoglobin, albumin-globulin ratio, lactate dehydrogenase, neutrophil-to-lymphocyte ratio, white blood cell and albumin^3–7^. However, the most studies were conducted with small samples and their results were inconsistent. The albumin-to-alkaline phosphatase ratio (AAPR), which has recently been proved to be a significantly prognostic predictor for hepatocellular carcinoma and metastatic nasopharyngeal carcinoma, but it has not yet been studied in UTUC patients^8,9^.

Therefore, the present study focused on the prognostic role of preoperative AAPR in UTUC patients undergoing RNU in our center.

## Material and Methods

### Patients

**Figure 1:**
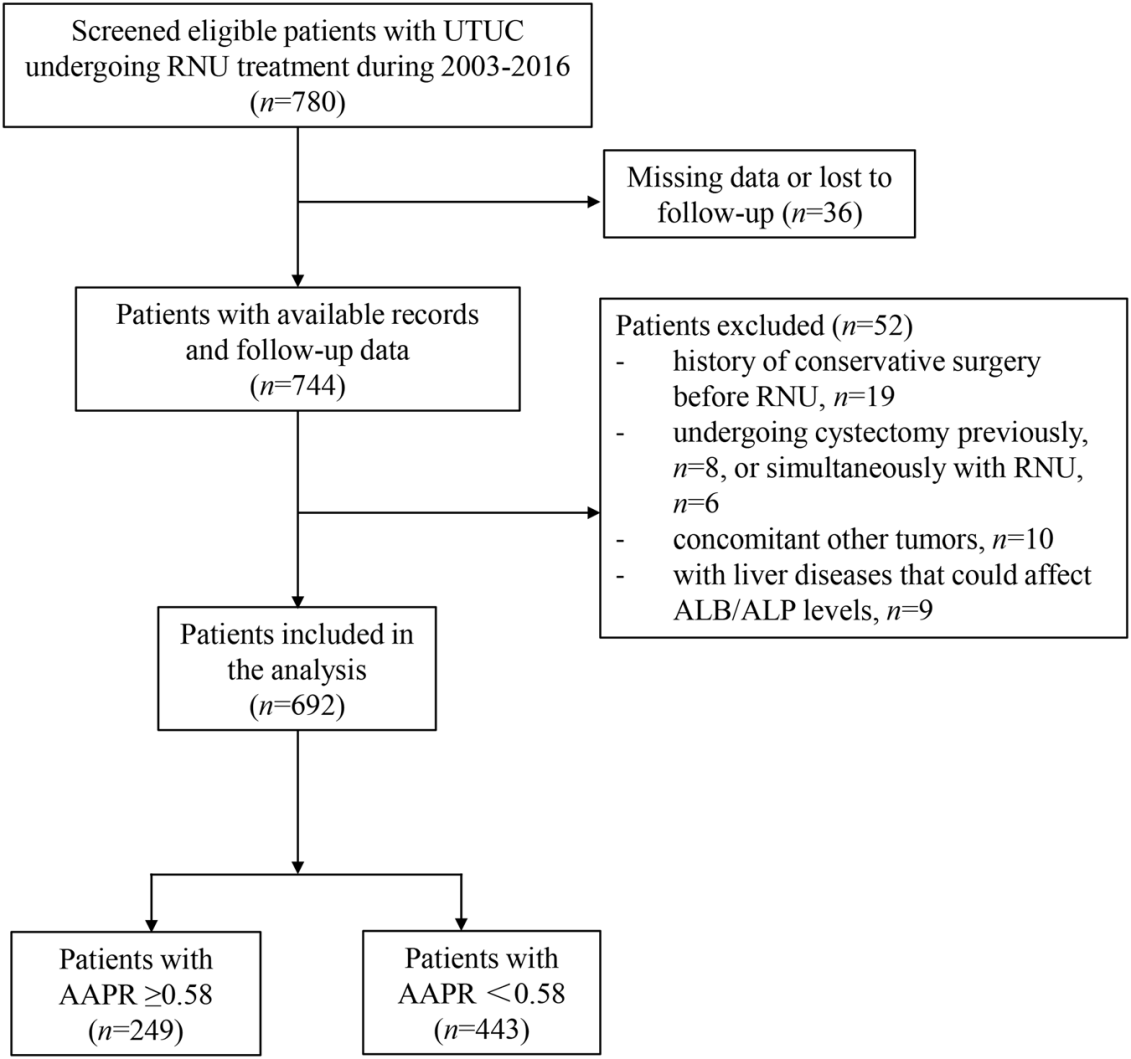
The patient selection flowchart.

The data of 780 patients diagnosed with UTUC between 2003 and 2016 were retrospectively gathered at our center. Only patients received RNU were included in this study, thus 19 patients who received conservative treatments before RNU were excluded. Thirty-six patients were excluded from the present study as a result of missing data. In addition, patients with the previous cystectomy for invasive bladder cancer (n = 8), patients underwent RNU plus radical cystectomy (n = 6) and patients with concomitant non-urothelial carcinomas (n = 10) and those with liver diseases that could affect ALB or ALP levels (n = 9) were also excluded; none patients received neo-adjuvant chemotherapy before surgery. (Fig. 1) The study was approved by the Ethics Committee of West China Hospital and the methods were carried out in accordance with the approved guidelines. For this type of study, informed consent is not required.

### Methods

The methods have been fully described in our previous papers^10,11^. Simply, all RNU specimens were respectively evaluated by two pathologists according to standard procedures. Baseline clinicopathological features and laboratory assessments within 30 days before RNU including hemoglobin, ALB and ALP, were obtained from the hospital database. The receiver-operating characteristic (ROC) curve and Youden index (Youden index = sensitivity + specificity-1) were applied to select the cut-off value of AAPR^12^. The AAPR of 0.58 was chosen as the threshold value as it had the maximum Youden index value.

Postoperative surveillance was performed according to the recommendation of EAU guideline^1^. In simple term, physical examination, blood laboratory tests and chest radiography were performed at every visiting; cystoscopy and urinary cytology were done at 3 month, and then annually for at least 5 years. Chest/abdomen CT/MRI were performed every 6 month for 2 years, and then annually to detect any postoperative recurrence or metastasis. If necessary, other tests such as bone scan can be applied.

### Statistical Analysis

The Pearson’s chi-squared test and Student’s t-test were utilized to assess the categorical variables and continuous variables, respectively. The relationship between AAPR and clinicopathological features were analyzed by logistic regression analyses. The time-to-event analyses were performed using Kaplan–Meier method, and the log-rank test was applied to compare distributions. To identify the independent prognostic factors, univariate and multivariate Cox regression analyses were performed. Hazard ratios (HRs) with corresponding 95% confidence intervals (CIs) and two-side P values were reported. All variables that had a P value < 0.1 in univariate analysis were included in the multivariate model. A P <0.05 was considered to indicate statistical significance. The software SPSS version 22.0 (IBM Corp., Armonk, NY, USA) was applied for all statistical analyses in this study.

## Result

A total of 692 participants were finally included in the present study with a mean age of 65.8 ± 11.4 years old. The baseline characteristics of the cohort were shown in Table 1. Among them, 460 patients underwent open RNU and 232 patients had laparoscopic RNU. Only 3.4% (n = 23) of patients had the chronic kidney disease (CKD) stage >3. 22 (3.2%) patients had a history of bladder cancer and 74 (10.7%) patients were found with a concomitant carcinoma in the bladder. The patients were divided into two groups according to their AAPR value < or ≥0.58 (AAPR <0.58, n = 443 and AAPR ≥0.58, n = 249). There was no difference between two groups in age, gender, tumor side and location, surgical approaches, renal function, multifocaity and adjuvant therapy. While, the logistic regression analyses found that lower AAPR was associated with advanced tumor stage (P < 0.001; RR = 1.778 for T3 and RR = 2.941 for T4) and high tumor grade (P = 0.010; RR = 1.574), concomitant variant histology (CVH) (P = 0.013; RR = 1.634) and anemia (P = 0.001; RR = 1.707) as well as larger tumor size (P = 0.020; RR = 1.476).

**Table 1:** The relationship between AAPR and clinicalpathological parameters in the present cohort (n = 692).

Variables	Total (n = 692)	AAPR <0.58 (n = 443, 64.0%)	AAPR ≥0.58 (n = 249, 36.0%)	*P*
Gender (Male vs Female)	398/294	246/197	152/97	0.173
Age (>67 vs ≤67 years)	341/351	211/130	130/119	0.268
Smoking (Yes vs No)	197/495	120/323	77/172	0.293
Tumor side (Right vs Left)	336/356	206/237	130/119	0.155
Surgical approach				0.210
Open RNU	460(66.5)	302(68.2)	158(63.5)	
Laparoscopic RNU	232(33.5)	141(31.8)	91(36.5)	
CKD stage				0.748
CKD 1	109(15.8)	74(16.7)	35(14.1)	
CKD 2	304(43.9)	191(43.1)	113(45.4)	
CKD 3	256(37.0)	162(36.6)	94(37.8)	
CKD4-5	23(3.4)	16(3.6)	7(2.8)	
Hydronephrosis (Yes vs No)	427/265	276/167	151/98	0.684
Anemia (Yes vs No)	278/414	198/245	80/169	0.001
Tumor location				0.488
Pelvicalyceal	372(53.8)	241(54.4)	131(52.6)	
Ureteric	199(28.8)	121(27.3)	78(31.3)	
Both	121(17.5)	81(18.3)	40(16.1)	
Tumor stage, *n* (%)				<0.001
Tis, Ta, T1	211(30.5)	116(26.2)	95(38.2)	
T2	139(20.1)	83(18.7)	56(22.5)	
T3	241(34.8)	165(37.2)	76(30.5)	
T4	101(14.6)	79(17.8)	22(8.8)	
Tumor grade, *n* (%)				0.011
Low	180(26.0)	101(22.8)	79(31.7)	
High	512(74.0)	342(77.2)	170(68.3)	
Lymph node status, *n* (%)				0.008
pN0	84(12.1)	56(12.6)	28(11.2)	
pNx	541(78.2)	333(75.2)	208(83.5)	
pN+	67(9.7)	54(12.2)	13(5.2)	
Lymph node resection, *n* (%)	151(21.8)	110(24.8)	41(16.4)	0.011
LVI (Positive vs Negative)	104/588	68/375	36/213	0.825
Tumor size (>3 vs ≤3 cm)	469/223	314/129	155/94	0.022
Surgical margin status (Positive vs Negative)	54/638	39/404	15/234	0.237
Multifocality (Present vs Absent)	113/579	65/378	48/201	0.133
CVH (With vs Without)	159/533	115/328	44/205	0.014
Bladder cancer status, *n* (%)				0.126
No	596(86.1)	385(86.9)	211(84.7)	
Previous	22(3.2)	17(3.8)	5(2.0)	
Concomitant	74(10.7)	41(9.3)	33(13.3)	
Adjuvant therapy (Yes vs No)	285/407	171/272	114/135	0.077
Albumin (Mean ± SD)	39.82 ± 4.82	39.14 ± 4.76	41.06 ± 4.68	<0.001
ALP (Mean ± SD)	81.05 ± 27.89	92.71 ± 27.5	60.30 ± 12.07	<0.001

^*^Note: AAPR: Albumin-to-Alkaline Phosphatase Ratio; CVH, concomitant variant histology; LVI, lymphovascular invasion; RNU, radical nephroureterectomy.

**Figure 2:**
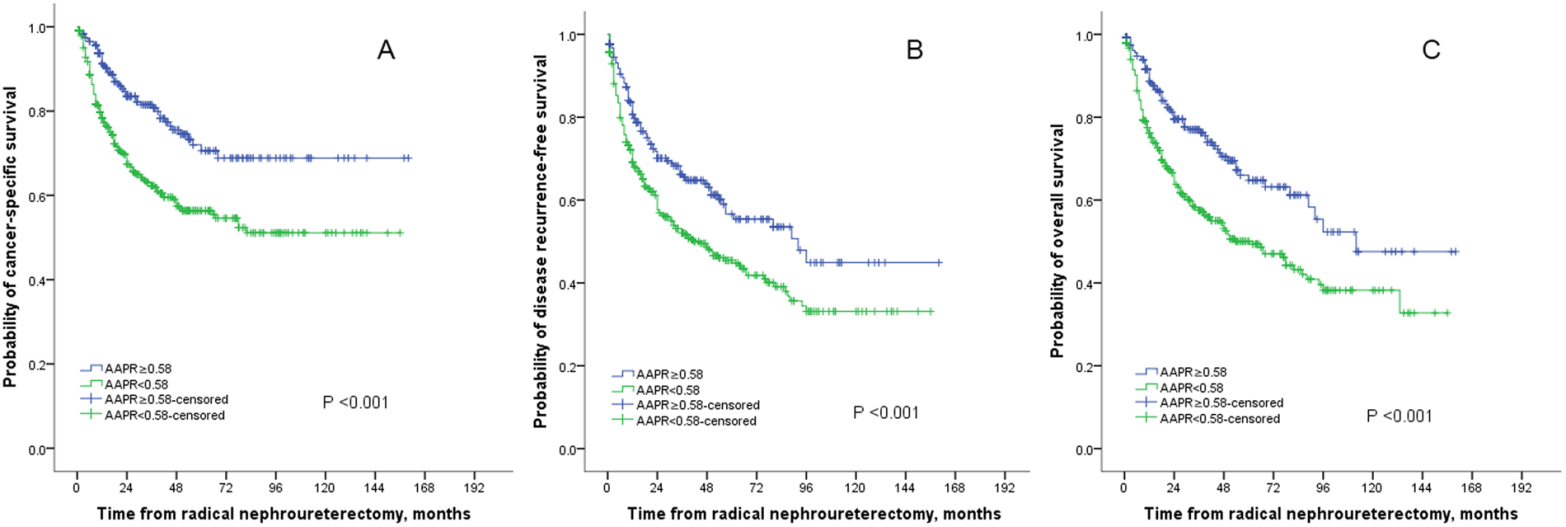
Kaplan–Meier curves for CSS (**A**), RFS (**B**) and OS (**C**) stratified according to APPR value in patients undergoing RNU of UTUC.

**Figure 3:**
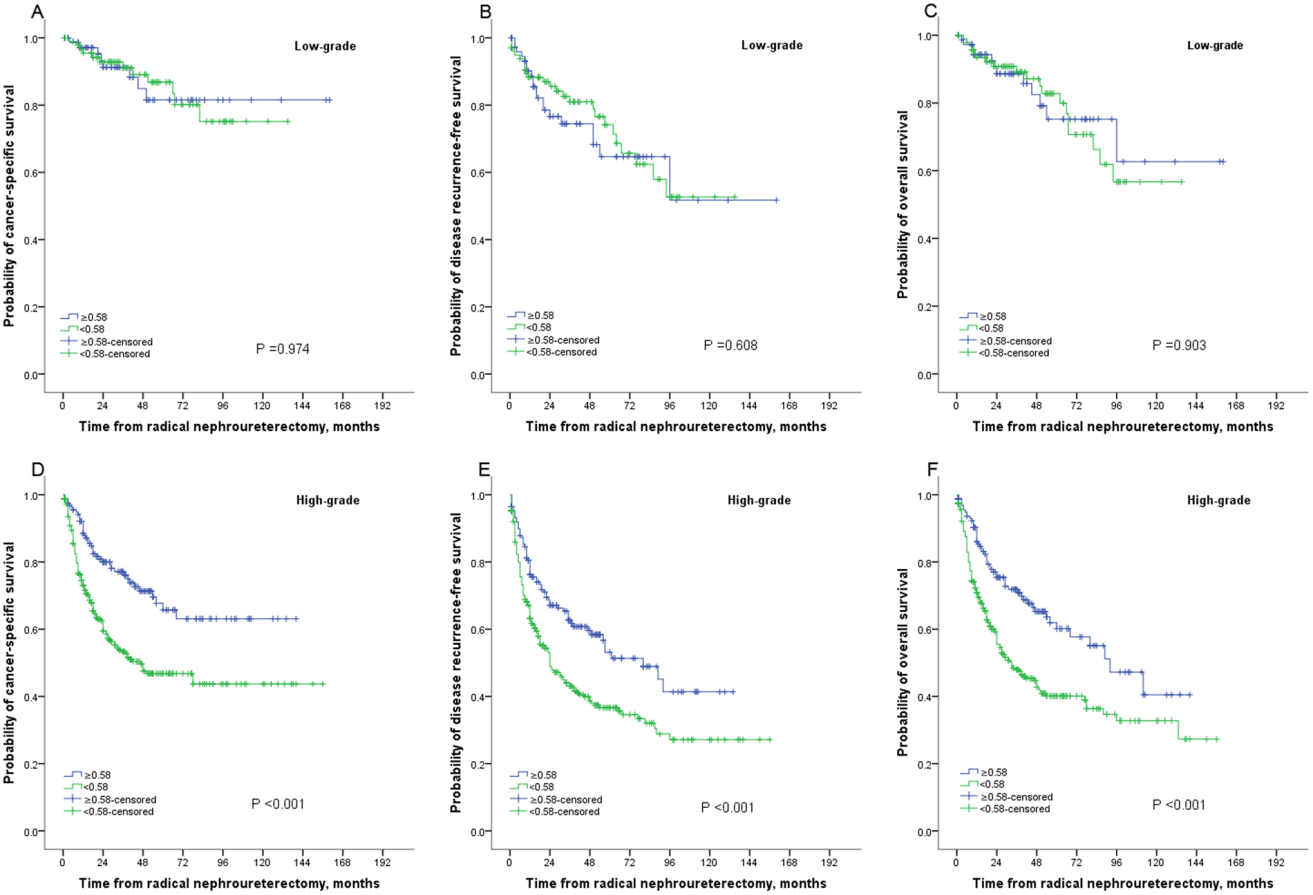
Kaplan–Meier curves for survival outcomes stratified according to APPR value in patients with low- or high-grade disease.

The median follow-up duration was 42 (IQR: 20–75) months. At the last follow-up, 249 patients died of all-causes and 199 patients died of UTUC. The 5-year overall survival (OS), cancer-specific survival (CSS), and disease recurrence-free survival (RFS) were 50.0%, 56.3%, 45.4% in patients with AAPR <0.58, and 64.8%, 70.6%, and 56.6%, respectively, in their counterparts. Kaplan–Meier curves showed that AAPR <0.58 was significantly associated with worse OS, CSS and RFS (all P < 0.001; see Fig. 2A–C). In addition, Kaplan–Meier curves suggested that AAPR <0.58 was also significantly related to higher mortality in subgroup with high-grade UTUC; however, in patients with low-grade disease, there was no difference between two groups (Fig. 3A–F). ROC curves found that AAPR had the higher AUC (area under curve) values for OS (AAPR 0.577, ALB 0.519, ALP 0.548), CSS (AAPR 0.583, ALB 0.520, ALP 0.570), and RFS (AAPR 0.557, ALB 0.502, ALP 0.540).

Univariate analysis found the AAPR <0.58 was associated with significantly inferior OS (HR 1.823, 95%CI: 1.374–2.419; P < 0.001), CSS (HR 2.026, 95%CI: 1.464–2.803; P < 0.001) and RFS (HR 1.550, 95%CI: 1.202–1.999; P = 0.001) (Table 2). Also, the pT stage, tumor grade, lymph node metastasis, lymphovascular invasion, CVH, tumor size, positive surgical margin, and anemia were significantly related to survival outcomes. After adjustment for preoperative clinical features, the results of multivariate analysis showed that the pretreatment AAPR was an independent risk factor for OS (HR 1.587, 95%CI: 1.185–2.126; P = 0.002), CSS (HR 1.746, 95%CI: 1.249–2.440; P = 0.001), and RFS (HR 1.337, 95%CI: 1.027–1.739; P = 0.031). Other factors, including pT stage, tumor grade, lymph node metastasis, CVH, tumor size, and anemia were also determined to be independent prognostic predictors in multivariate analysis (Table 3).

**Table 2:** Univariable regression analysis of clinicopathological parameters for the prediction of survival outcomes in UTUC patients.

Variables	Overall survival			Cancer-specific survival			Disease recurrence-free survival		
	HR	95%CI	*P*	HR	95%CI	*P*	HR	95%CI	*P*
Age (>67 years vs ≤67 years)	1.034	0.807–1.325	0.789	0.944	0.716–1.245	0.683	0.945	0.750–1.190	0.629
Sex (Male vs Female)	0.891	0.695–1.143	0.364	0.828	0.628–1.093	0.183	0.869	0.689–1.095	0.235
Smoking (yes vs no)	0.904	0.681–1.199	0.483	0.851	0.618–1.172	0.324	0.885	0.680–1.152	0.364
Tumor site			0.818			0.738			0.667
Pelvicalyceal	1.000	Reference		1.000	Reference		1.000	Reference	
Ureteric	0.941	0.702–1.260	0.683	1.008	0.731–1.392	0.959	0.945	0.720–1.241	0.686
Both	1.064	0.753–1.503	0.724	1.157	0.793–1.688	0.448	1.112	0.809–1.529	0.512
Tumour stage			<0.001			<0.001			<0.001
Tis, Ta, T1	1.000	Reference		1.000	Reference		1.000	Reference	
T2 vs Tis, Ta, T1	1.659	1.041–2.644	0.033	1.716	0.990–2.974	0.054	1.518	1.008–2.284	0.046
T3 vs Tis, Ta, T1	3.572	2.428–5.255	<0.001	4.054	2.579–6.374	<0.001	3.022	2.152–4.245	<0.001
T4 vs Tis, Ta, T1	8.725	5.788–13.156	<0.001	10.558	6.581–16.939	<0.001	7.451	5.142–10.798	<0.001
Tumor grade (high vs low)	3.104	2.141–4.501	<0.001	3.835	2.440–6.029	<0.001	2.409	1.757–3.303	<0.001
Lymph node status			<0.001			<0.001			<0.001
pN0	1.000	Reference		1.000	Reference		1.000	Reference	
pNx vs pN0	1.484	0.960–2.295	0.076	1.498	0.904–2.482	0.117	1.496	1.001–2.237	0.050
pN + vs pN0	5.318	3.222–8.776	<0.001	6.055	3.452–10.621	<0.001	5.512	3.453–8.797	<0.001
LVI (positive vs negative)	2.628	1.970–3.507	<0.001	2.843	2.074–3.896	<0.001	2.309	1.750–3.047	<0.001
CVH (With vs Without)	2.176	1.671–2.833	<0.001	2.375	1.775–3.178	<0.001	1.997	1.554–2.565	<0.001
CKD4–5 vs CKD1–3	1.727	0.987–3.019	0.055	1.447	0.741–2.824	0.279	1.417	0.812–2.472	0.219
Size (>3 cm vs ≤3 cm)	2.008	1.502–2.685	<0.001	2.054	1.481–2.850	<0.001	1.871	1.435–2.441	<0.001
Margin status (positive vs negative)	2.251	1.536–3.299	<0.001	2.426	1.606–3.665	<0.001	1.979	1.362–2.875	<0.001
Multifocality (present vs absent)	0.929	0.658–1.313	0.677	1.008	0.694–1.464	0.967	0.957	0.696–1.316	0.789
Surgical approach (laparoscopic vs open)	0.726	0.540–0.974	0.033	0.675	0.487–0.936	0.019	0.877	0.676–1.138	0.323
Tumor side (right vs left)	1.062	0.829–1.360	0.635	1.098	0.833–1.448	0.507	1.069	0.848–1.346	0.573
AAPR (<0.58 VS ≥0.58)	1.823	1.374–2.419	<0.001	2.026	1.464–2.803	<0.001	1.550	1.202–1.999	0.001
Anemia (Yes vs No)	2.012	1.570–2.578	<0.001	2.067	1.567–2.728	<0.001	1.684	1.336–2.122	<0.001
Adjuvant therapy (yes vs no)	0.873	0.680–1.120	0.286	0.931	0.706–1.230	0.616	1.111	0.882–1.400	0.371

^*^Note: AAPR: Albumin-to-Alkaline Phosphatase Ratio; CVH, concomitant variant histology; LVI, lymphovascular invasion; RNU, radical nephroureterectomy.

**Table 3:** Multivariable Cox regression analyses of survival outcomes in patients with urinary tract urothelial carcinoma.

Variables	Overall survival			Cancer-specific survival			Disease recurrence-free survival		
	HR	95%CI	*P*	HR	95%CI	*P*	HR	95%CI	*P*
Tumour stage			<0.001			<0.001			<0.001
Tis, Ta, T1	1.000	Reference		1.000	Reference		1.000	Reference	
T2 vs Tis, Ta, T1	1.480	0.921–2.378	0.105	1.504	0.857–2.637	0.155	1.384	0.914–2.098	0.125
T3 vs Tis, Ta, T1	2.197	1.446–3.338	<0.001	2.399	1.467–3.921	<0.001	2.163	1.498–3.122	<0.001
T4 vs Tis, Ta, T1	3.429	2.080–5.653	<0.001	3.798	2.133–6.763	<0.001	3.560	2.251–5.630	<0.001
Tumor grade (high vs low)	2.065	1.385–3.080	<0.001	2.410	1.481–3.923	<0.001	1.682	1.198–2.362	0.003
Lymph node status			0.001			0.002			<0.001
pN0	1.000	Reference		1.000	Reference		1.000	Reference	
pNx vs pN0	1.939	1.241–3.029	0.004	1.973	1.179–3.301	0.010	1.907	1.267–2.871	0.002
pN+ vs pN0	2.653	1.556–4.524	<0.001	2.869	1.582–5.203	0.001	3.017	1.827–4.981	<0.001
LVI (positive vs negative)	1.271	0.918–1.760	0.148	1.286	0.902–1.833	0.165	1.072	0.780–1.473	0.669
CVH (With vs Without)	1.483	1.124–1.958	0.005	1.561	1.150–2.121	0.004	1.350	1.038–1.757	0.025
CKD4–5 vs CKD1-3	1.138	0.639–2.025	0.661	0.951	0.479–1.888	0.885	0.989	0.557–1.756	0.970
Size (>3 cm vs ≤3 cm)	1.578	1.159–2.149	0.004	1.496	1.057–2.117	0.023	1.491	1.128–1.972	0.005
Margin status (positive vs negative)	1.200	0.805–1.791	0.371	1.230	0.799–1.894	0.347	1.099	0.744–1.624	0.635
Surgical approach (laparoscopic vs open)	0.917	0.678–1.240	0.574	0.884	0.631–1.238	0.473		—	
AAPR (<0.58 VS ≥0.58)	1.587	1.185–2.126	0.002	1.746	1.249–2.440	0.001	1.337	1.027–1.739	0.031
Anemia (Yes vs No)	1.588	1.222–2.126	0.001	1.575	1.177–2.108	0.002	1.339	1.048–1.712	0.020

^*^Note: AAPR: Albumin-to-Alkaline Phosphatase Ratio; CVH, concomitant variant histology; LVI, lymphovascular invasion; RNU, radical nephroureterectomy.

Subgroup analyses found that the decreased AAPR was also proved to be independently associated with poor OS (HR 1.726, 95%CI: 1.252–2.380; P = 0.001), CSS (HR 1.906, 95%CI: 1.324–2.743; P = 0.001) and RFS (HR 1.509, 95%CI: 1.120–2.033; P = 0.007) in patients with high-grade UTUC; however in patients with low-grade disease, AAPR had a neutral role in OS (HR 0.905, 95%CI: 0.408–2.005, P = 0.805), CSS (HR 0.674, 95%CI: 0.240–1.895, P = 0.455), and RFS (HR 0.727, 95%CI:0.383–1.381, P = 0.273) (Supplementary Tables 1 and 2).

## Discussion

The prognosis of patients with UTUC remained unsatisfactory. Previous evidence had found that most of the patients died from UTUC within 1 year of disease recurrence and the probability of surviving 2 years after disease recurrence was only 20%. Thus, to stratify the patients with high risk of poor prognosis was important. However, to accurately stage patients before definitive therapy was unavailable, which limited the management of UTUC. A few retrospective studies had found that patients with UTUC could benefit from the usage of neoadjuvant chemotherapy^13,14^. Preoperative imaging could not accurately stage UTUC, even preoperatively endoscopic biopsy is rarely sufficient for the determination of the degree of microscopic invasion. Thus, preoperative prognostic markers would be valuable tools to improve the accuracy of risk stratification models.

Anthony *et al*. reported that AAPR was a powerful prognostic predictor with the highest C-index among liver biochemical parameters^8^. Then Nie *et al*. reported that AAPR was independently related to the prognosis of patients with metastatic NPC, and it has better predict ability than ALB or ALP alone^9^. In our study, we also found the AAPR had the higher AUC compared with ALB or ALP in UTUC patients, indicating that the AAPR better predicted their survival outcomes. Meanwhile, lower AAPR was related to advanced pT stage and high tumor grade, CVH and larger tumor size. Multivariate analysis found lower AAPR was independently associated with poor OS, CSS and RFS in patients with UTUC undergoing RNU.

The mechanism of AAPR in human malignancies including UTUC remains unclear. Increasing evidence shows that the presence of nutritional deficiencies and systematic inflammatory response might play an important role in the development and progress of human cancers and also be associated with inferior prognosis in patients undergoing resection for solid tumors^3,15^. ALB is a stable and flexible serum protein and modulates the systemic and organ inflammatory reaction, as well as exert antioxidant effects against carcinogens^16^. Also, low ALB reflected nutrient deficiency exits which could decrease immune function and lead to poor anti-cancer response^17^. Recently, evidence has found that ALB was a useful prognostic predictor in various malignancies such as hepatocellular carcinoma (HCC), renal carcinoma, prostate cancer, and colorectal cancer as well as UTUC^3,4,18–20^. ALP is a hydrolase enzyme found primarily in the liver, bile duct, kidney, bone, and placenta. Serum ALP will increase during some pathological conditions, such as HCC, kidney disease and bone metastasis^21,22^. In addition, ALP has been found to be an independent risk factor for survival outcomes in patients with HCC and nasopharyngeal carcinoma (NPC)^23,24^. Although Kluth *et al*. did not find the prognostic value of ALP in patients with UTUC, Sheth *et al*. reported that ALP ≥116 U/l was related to multiple adverse clinicopathological parameters and poor RFS in high-grade UTUC^4,5^.

Interestingly, our results showed that the decreased AAPR contributed to worse survival outcomes in high-grade UTUC; but in terms of low-grade disease, there was no relationship between them. To date, according to the available studies, only few studies had discussed the roles of ALP or ALB in different subgroups in various malignancies, and also their results were inconsistent. Some found they could be used as prognostic markers in patients with non-metastatic diseases, while others recommended they was significantly useful only in those with advanced diseases or with metastatic diseases^4,25^. Some researchers suggested the dynamic change of ALP or ALB might be related to bone or liver metastasis or metastatic tendency, which may partly explain why low AAPR could contribute to poor prognosis in high-grade UTUC with high metastatic and aggressive ability. Specifically, due to the small sample of low-grade disease and relatively short follow-up duration, our study did not found any significant prognostic predictors in this population. Therefore, the conclusions from patients with low-grade UTUC should be applied with caution and should be validated by future studies with larger samples and longer follow-up time.

Limitations of this study should be described. First, our cohort was retrospectively included which may cause a selection bias. Also, the adjuvant chemotherapies and radiotherapies were variously administered, which may affect the patients‘ outcomes differently. As the additional benefits of the lymphadenectomy pattern for UTUC were not clear to date, thus lymphadenectomy was not routinely performed which may affect the survival outcomes. Moreover, other potential prognostic inflammatory markers such as C-reactive protein, fibrinogen, and cytokines were not available due to limited data which may add additional prognostic value to the model. Despite limitations above, the present study was the first one with a large sample to assess the prognostic impact of AAPR on patients with UTUC after RNU.

## Conclusions

The AAPR was a novel derived indicator from routinely available tests. In the present cohort, we demonstrated that lower AAPR was a superior indicator than ALB and ALP, and was independently associated with inferior survival outcomes in UTUC patients. Using the AAPR for subclassification of high-grade UTUC seems to further identify a poor prognostic group and contribute to clinical decisions making. Future prospective studies should be performed to validate its prognostic role.

## Electronic supplementary material

Supplementary table 1–2
