# Supplementary material for: The prognostic significance of Albumin-to-Alkaline Phosphatase Ratio in upper tract urothelial carcinoma
Source: Sci Rep. 2018 Aug 17;8:12311. doi: 10.1038/s41598-018-29833-5 (PMC6097991; doi:10.1038/s41598-018-29833-5)
Supplement: Supplementary file 1 — Supplementary table 1–2 [file 41598_2018_29833_MOESM1_ESM.docx]

Supplementary file

The prognostic significance of Albumin-to-Alkaline Phosphatase Ratio in upper tract urothelial carcinoma

Ping Tan ^a, #^, Nan Xie ^b, #^, Jianzhong Ai ^a, #^, Hang Xu ^a^, Huan Xu ^c^, Liangren Liu ^a^, Lu Yang ^a, *^, Qiang Wei ^a,^ *

^a^ Department of Urology, Institute of Urology, West China Hospital, Sichuan University, Chengdu, Sichuan.

^b^ Department of Emergency, West China Hospital, Sichuan University, Chengdu

^c^ Department of Pathology, West China Hospital, Sichuan University, Chengdu

* Correspondence to Lu Yang and Qiang Wei. Department of Urology, Institute of Urology, West China Hospital, Sichuan University, Number 37, Guoxue Alley, Chengdu, Sichuan 610041, China. E-mail: [wycleflue@163.com](mailto:wycleflue@163.com) (Lu Y); [weiqiang933@126.com](mailto:weiqiang933@126.com) (Qiang W). Tel: +86-189-806-014-25.

^#^ Authors contributed equally to this work.

Supplementary Table 1. Multivariable Cox regression analyses of survival outcomes in patients with low-grade disease.

| Variables | Overall Survival | | | Cancer-specific survival | | | Recurrence-free survival | | |
| --- | --- | --- | --- | --- | --- | --- | --- | --- | --- |
|  | HR | 95%CI | *P* | HR | 95%CI | *P* | HR | 95%CI | *P* |
| Tumour stage |  |  | 0.650 |  |  | 0.124 |  |  | 0.136 |
| Tis, Ta, T1 | 1.000 | Reference |  | 1.000 | Reference |  | 1.000 | Reference |  |
| T2 vs Tis, Ta, T1 | 1.155 | 0.439-3.041 | 0.771 | 0.493 | 0.122-1.995 | 0.321 | 1.104 | 0.494-2.467 | 0.809 |
| T3 vs Tis, Ta, T1 | 1.376 | 0.496-3.820 | 0.540 | 1.170 | 0.342-4.005 | 0.802 | 2.127 | 0.993-4.555 | 0.052 |
| T4 vs Tis, Ta, T1 | 2.909 | 0.545-15.528 | 0.212 | 5.079 | 0.903-28.579 | 0.065 | 3.715 | 0.743-18.570 | 0.110 |
| Lymph node status |  |  | 0.811 |  |  | 0.865 |  |  | 0.684 |
| pN0 | 1.000 | Reference |  | 1.000 | Reference |  | 1.000 | Reference |  |
| pNx vs pN0 | 1.170 | 0.342-4.004 | 0.802 | 0.706 | 0.193-2.580 | 0.598 | 1.652 | 0.497-5.493 | 0.413 |
| pN+ vs pN0 | 2.564 | 0.148-44.380 | 0.518 | 0.889 | 0.051-15.570 | 0.936 | 2.266 | 0.147-35.000 | 0.558 |
| LVI (positive vs negative) | 1.192 | 0.135-10.532 | 0.874 | 2.099 | 0.210-21.007 | 0.528 | 0.648 | 0.079-5.309 | 0.686 |
| CVH (With vs Without) | 1.150 | 0.422-3.133 | 0.785 | 2.212 | 0.745-6.567 | 0.153 | 1.376 | 0.616-3.077 | 0.436 |
| CKD4-5 vs CKD1-3 | - |  |  | - |  |  | - |  |  |
| Size (>3cm vs ≤3cm) | 1.434 | 0.615-3.340 | 0.404 | 1.428 | 0.490-4.161 | 0.514 | 1.278 | 0.656-2.492 | 0.471 |
| Margin status (positive vs negative) | - |  |  | - |  |  | - |  |  |
| Surgical approach (laparoscopic vs open) | 1.817 | 0.761-4.340 | 0.179 | 0.908 | 0.296-2.788 | 0.867 | 1.463 | 0.741-2.886 | 0.273 |
| AAPR (＜0.58 VS ≥0.58) | 0.905 | 0.408-2.005 | 0.805 | 0.674 | 0.240-1.895 | 0.455 | 0.727 | 0.383-1.381 | 0.273 |
| Anemia (Yes vs No) | 1.286 | 0.539-3.065 | 0.571 | 0.931 | 0.315-2.753 | 0.897 | 0.913 | 0.432-1.931 | 0.812 |

*Note: AAPR: Albumin-to-Alkaline Phosphatase Ratio; CVH, concomitant variant histology; LVI, lymphovascular invasion; RNU, radical nephroureterectomy.

Supplementary Table 2. Multivariable Cox regression analyses of survival outcomes in patients with high-grade disease.

| Variables | Overall Survival | | | Cancer-specific survival | | | recurrence-free survival | | |
| --- | --- | --- | --- | --- | --- | --- | --- | --- | --- |
|  | HR | 95%CI | *P* | HR | 95%CI | *P* | HR | 95%CI | *P* |
| Tumour stage |  |  | <0.001 |  |  | <0.001 |  |  | <0.001 |
| Tis, Ta, T1 | 1.000 | Reference |  | 1.000 | Reference |  | 1.000 | Reference |  |
| T2 vs Tis, Ta, T1 | 1.579 | 0.892-2.794 | 0.117 | 1.936 | 0.996-3.760 | 0.051 | 1.528 | 0.920-2.537 | 0.101 |
| T3 vs Tis, Ta, T1 | 2.486 | 1.516-4.077 | <0.001 | 2.916 | 1.611-5.277 | <0.001 | 2.341 | 1.503-3.647 | <0.001 |
| T4 vs Tis, Ta, T1 | 3.664 | 2.075-6.470 | <0.001 | 4.363 | 2.235-8.517 | <0.001 | 3.783 | 2.236-6.400 | <0.001 |
| Lymph node status |  |  | 0.003 |  |  | 0.002 |  |  | <0.001 |
| pN0 | 1.000 | Reference |  | 1.000 | Reference |  | 1.000 | Reference |  |
| pNx vs pN0 | 2.008 | 1.244-3.241 | 0.004 | 2.222 | 1.264-3.907 | 0.006 | 1.891 | 1.218-2.936 | 0.005 |
| pN+ vs pN0 | 2.610 | 1.494-4.561 | 0.001 | 3.101 | 1.641-5.862 | <0.001 | 2.909 | 1.728-4.900 | <0.001 |
| LVI (positive vs negative) | 1.296 | 0.930-1.805 | 0.126 | 1.308 | 0.912-1.875 | 0.144 | 1.113 | 0.802-1.545 | 0.523 |
| CVH (With vs Without) | 1.505 | 1.124-2.015 | 0.006 | 1.527 | 1.108-2.104 | 0.010 | 1.338 | 1.010-1.774 | 0.043 |
| CKD4-5 vs CKD1-3 | 1.159 | 0.649-2.067 | 0.618 | 0.961 | 0.483-1.911 | 0.910 | 1.027 | 0.577-1.830 | 0.927 |
| Size (>3cm vs ≤3cm) | 1.586 | 1.134-2.219 | 0.007 | 1.520 | 1.047-2.205 | 0.028 | 1.547 | 1.132-2.114 | 0.006 |
| Margin status (positive vs negative) | 1.216 | 0.814-1.815 | 0.340 | 1.236 | 0.803-1.902 | 0.335 | 1.103 | 0.746-1.633 | 0.623 |
| Surgical approach (laparoscopic vs open) | 0.837 | 0.603-1.161 | 0.286 | 0.863 | 0.604-1.231 | 0.416 | 1.024 | 0.760-1.380 | 0.876 |
| AAPR (＜0.58 VS ≥0.58) | 1.726 | 1.252-2.380 | 0.001 | 1.906 | 1.324-2.743 | 0.001 | 1.509 | 1.120-2.033 | 0.007 |
| Anemia (Yes vs No) | 1.607 | 1.214-2.126 | 0.001 | 1.608 | 1.182-2.187 | 0.002 | 1.420 | 1.087-1.853 | 0.010 |

*Note: AAPR: Albumin-to-Alkaline Phosphatase Ratio; CVH, concomitant variant histology; LVI, lymphovascular invasion; RNU, radical nephroureterectomy.
